# Detection of dissociative states in patients during the administration of Es-ketamine by monitoring with the Dissociative Experience Scale II (DES II) - Part I

**DOI:** 10.1192/j.eurpsy.2025.414

**Published:** 2025-08-26

**Authors:** C. Serritella, F. Ferraiuolo, M. Piccirillo, E. Lastrucci, A. Frasso, G. De Mattia, T. Salvati, D. Riccio

**Affiliations:** 1 Università degli studi di Napoli Federico II, Psichiatria; 2 Seconda Università degli Studi di Napoli (SUN), Psichiatra, psicoterapeuta Cognitivo-Costruttivista, Napoli; 3 Università degli studi di Potenza, Scienze biostatistiche, Potenza; 4 ASL Caserta, UOSM 12, Psicologa, Caserta; 5 Università degli studi Suor Orsola Benincasa, Professoressa, Psicoterapeuta, Napoli; 6 Università Telematica Pegaso, Linguista, Milano, Italy

## Abstract

**Introduction:**

The effectiveness of Es-ketamine therapy in the treatment of psycho-pathological symptoms is the subject of growing interest in the scientific community. Through the use of validated psychometric instruments, this study aims to delineate more precisely the mechanisms of action and clinical potential of this therapy, contributing to the advancement of knowledge in psychiatry.

**Objectives:**

The main objective of the present study is to analyses in depth and systematically the onset and development of the dissociative state, as well as the different forms of dissociation, in patients undergoing treatment with Es-ketamine (Spravato). Particular attention will be paid to the distinction between drug-induced dissociation and dissociation of a psychotic nature.

**Methods:**

Two psychometric instruments were used: the Clinical Global Impression (CGI) and the Dissociative Experience Scale II (DES II). The CGI assessed symptom severity (Severity of Illness, SI), the overall clinical improvement (Global Improvement, GI) and treatment effectiveness with respect to side effects (Efficacy Index, EI). DES II measured the frequency and intensity of dissociative symptoms, considering a score of ≥30 as pathological. The initial sample of 16 patients was reduced to 15 (6 males and 9 females) due to the exclusion of one participant due to incomplete data.

**Results:**

Preliminary results indicate that treatment with Es-ketamine led to significant clinical improvement in the majority of patients. Quantitative analysis showed an improvement between the initial (T0) and subsequent (T1) assessment on both scales used. In the DES II, a mean effect of +0.98 was found, with a cumulative reduction of 14.65 points, indicating a decrease in dissociative symptomatology. In the CGI, the mean improvement presented an effect size of +56.57, with a range from +30.4 to +117.9, suggesting significant variability in treatment response. In two patients, a 31-year-old man and a 19-year-old woman, a clinical worsening associated with high levels of dissociation was observed, highlighting the potential role of factors such as age and dissociative vulnerability in treatment response.

**Conclusions:**

The CGI and DES II scales proved effective in monitoring clinical changes in patients treated with Es-ketamine, highlighting their complementarity. This study represents the first attempt to differentiate between traumatic and psychotic dissociation in Es-ketamine-treated patients, suggesting new opportunities to tailor therapeutic interventions therapeutic interventions. Further studies with larger samples and more robust methodological designs are needed to confirm the preliminary results, as well as to investigate the long-term impact of Esketamine on dissociation and the potential associated risks.

**Disclosure of Interest:**

None Declared

